# ﻿Two new species of *Phaeoclavulina* (Gomphaceae, Gomphales) from North China based on morphological and phylogenetic analysis

**DOI:** 10.3897/mycokeys.120.138950

**Published:** 2025-07-29

**Authors:** Xin Tong, Yue Gao, Hai-Qi Wang, Hao Zhou, Cheng-Lin Hou

**Affiliations:** 1 Department of Life Sciences, Natural History Museum of China, Tianqiaonandajie 126, Dongcheng, 100050, Beijing, China Natural History Museum of China Beijing China; 2 College of Life Science, Capital Normal University, Haidian, 100048, Beijing, China Capital Normal University Beijing China

**Keywords:** Clavarioid fungi, new taxa, systematics, taxonomy

## Abstract

Two new species, *Phaeoclavulinaaurea* and *P.fulva*, were discovered and described from North China. Identification was based on morphological observations combined with phylogenetic analysis of nrITS–nrLSU sequences. *Phaeoclavulinaaurea* is characterized by its pale yellow to golden yellow basidiomata, brownish black coloration with dark tips at maturity, and basidiospores bearing truncate spines. *Phaeoclavulinafulva* is distinguished by its dirty orange basidiomata, pale yellow stipe surface, and basidiospores ornamented with rounded warts. This study enriches the species diversity of *Phaeoclavulina* in North China.

## ﻿Introduction

*Phaeoclavulina*Brinkmann (1897: 197) (Gomphaceae, Gomphales), with *P.macrospora*Brinkmann (1897: 198) as the type species, was established in 1897 (Brinkmann 1897). It includes gomphoid and ramarioid forms characterized by ramarioid, unipileate, or merismatoid basidiomata. When pileate, basidiomata are glabrous or subtomentose and infundibuliform or flabelliform (González-Ávila et al. 2020). The genus exhibits a diverse palette of colors on the pileus and branch surfaces, varying from white, brown-green, pale to sordid olivaceous, violet, brown-yellow, and red cinnamon, through to gray, brick red, and pale to dark orange-yellow, even blue-green. Spores show the presence of clamps and are echinulate to verrucose, subreticulate, or reticulate (Giachini 2004; Giachini and Castellano 2011). Some *Phaeoclavulina* species are ectomycorrhizal. For example, *P.abietina* (Pers.) Giachini (2011: 189) is associated with *Betula*, *Pinus*, and *Pseudotsuga* (Herrera et al. 2002; Norvell and Exeter 2004); *P.flaccida* (Fr.)Giachini (2011: 192) with *Pinus* and *Quercus* (Kim et al. 2003); *P.zippelii* (Lév.) Overeem (1923: 262) with *Acacia*, *Casuarina*, and *Eucalyptus* (Sims et al. 1997); and *P.cyanocephala* (Berk. & M.A. Curtis) Giachini (2011: 191) with *Abies* (Estrada-Torres 1994).

Initially, Brinkmann (1897) placed *Phaeoclavulina* within the family Clavariaceae, along with other genera, viz. *Clavaria* Vaill. ex L. (1753: 1182), *Clavariella* P. Karst. (1881: 21), *Clavulina* J. Schröt. (1888: 442), and *Typhula* (Pers.) Fr. (1818: 296). Overeem (1923) recognized *Phaeoclavulina* as a distinct genus and assigned one species, *P.zippelii*, to it, which had previously been classified under *Clavaria* based on macromorphological characteristics. Despite this, the recognition of *Phaeoclavulina* did not gain widespread support among mycologists, and species of this genus were often accommodated with other ramarioid fungi having ochre spores in *Ramaria* Fr. ex Bonord. (1851: 166) (González-Ávila et al. 2020). Corner (1970) went further by placing the ramarioid species of *Phaeoclavulina* within Ramariasubgen.EchinoramariaCorner (1970: 238), a classification later adopted by other authors such as Marr and Stuntz (1973) and Petersen (1981). In recent research, *Phaeoclavulina* was once again recognized as a valid genus based on morphological, molecular, and phylogenetic data (Giachini 2004; Giachini et al. 2010; Giachini and Castellano 2011). It now includes not only the original species but also some gomphoid species with spiny, verrucose, subreticulate, or reticulate spores and a terrestrial and/or lignicolous substrate affinity (González-Ávila et al. 2020). Currently, approximately 57 species of *Phaeoclavulina* have been described from temperate and tropical ecosystems; however, it is likely more abundant in the tropics and subtropics (González-Ávila et al. 2020; Liu et al. 2022).

In China, 22 *Phaeoclavulina* species have been reported based on morphological features and phylogenetic analyses, viz. *P.abietina*, *P.aeruginea* P. Zhang (2022: 31), *P.bicolor* P. Zhang & W.H. Liu (2024: 6), *P.campestris* (K. Yokoy. & Sagara) Giachini (2011: 190), *P.capucina* (Pat.) Giachini (2011: 190), *P.cinnamomea* W.Q. Qin (2022: 32), *P.cokeri* (R.H. Petersen) Giachini (2011: 190), *P.curta* (Fr.) Giachini (2011: 190), *P.cyanocephala*, *P.decolor* (Berk. & M.A. Curtis) Giachini (2011: 191), *P.echinoflava* P. Zhang & W.H. Liu (2024: 7), *P.eumorpha* (P. Karst.) Giachini (2011: 191), *P.flaccida*, *P.grandis* (Corner) Giachini (2011: 193), *P.jilinensis* P. Zhang & W.H. Liu (2024: 9), *P.longicaulis* (Peck) Giachini (2011: 193), *P.macrospora*, *P.mutabilis* (Schild & R.H. Petersen) Giachini (2011: 194), *P.sikkimia* (S.S. Rattan & Khurana) Giachini (2011: 194), *P.viridis* (Pat.) Giachini (2011: 195), *P.yunnanensis* W.H. Lu, D.G. Zheng, Karun. & Tibpromma (2024: 113), and *P.zippelii* (Liu et al. 2022; Deng et al. 2024; Zheng et al. 2024). Despite numerous prior reports from China, there remains significant potential for the discovery of new species, particularly in North China.

Recently, several *Phaeoclavulina*-like samples were collected during an investigation of the Yanshan Mountains (39°40′–41°20′ N, 115°–119°47′ E) in North China, a warm temperate region. In this study, two new species are described and illustrated. The nuclear ribosomal internal transcribed spacer (nrITS) and the large subunit of nuclear ribosomal RNA (nrLSU) were sequenced from dried basidiomata of each species for phylogenetic analysis.

## ﻿Materials and methods

### ﻿Collecting and site description

The specimens were collected from Beijing and Tianjin, North China, between 2019 and 2023, and important collection data were recorded (Rathnayaka et al. 2024). These regions have a warm temperate continental monsoon climate, which supports a diverse assemblage of plant species (Zhou et al. 2022). Deciduous broad-leaved forest and mixed coniferous and broad-leaved forest are the dominant vegetation types in the area. Notable plant species include *Abiesnephrolepis* (Trautv.) Maxim., *Betulaplatyphylla* Suk., *Pinustabuliformis* Carr., *Populustomentosa* Carrière, and *Quercusmongolica* Fisch. ex Ledeb. (Wang et al. 2021; Zhou et al. 2022). The annual precipitation is approximately 700 mm (Zhou et al. 2022), and the elevation ranges from 200 to 2200 m. The collected specimens were dehydrated using an electric dryer (Dorrex) at 50 °C and then deposited in the Herbarium of the College of Life Sciences, Capital Normal University, Beijing, China (**BJTC**), and the Herbarium of the Natural History Museum of China, Beijing, China (**NNHMC**).

### ﻿Morphological observation

Macroscopic characteristics of the specimens were recorded, including basidiomata color, size, branching pattern, stipe color, morphology, and dimensions. Microscopic features were analyzed by examining thin sections mounted in 3% potassium hydroxide (KOH) or sterilized water. The morphology and dimensions of microscopic structures were observed and recorded using a light microscope (Olympus DP71, Tokyo, Japan). In the description of basidiospores, the abbreviation *n/m/p* indicates that *n* basidiospores were measured from *m* basidiomata of *p* collections. Measurements and *Q* values are presented in the format (a)b–c(d), where “a” represents the minimum value, “b–c” the 10% to 90% range, and “d” the maximum value. *Q* represents the ratio of basidiospore length to width in side view (Liu et al. 2022), and *Q_m_* represents the average *Q* value of all measured basidiospores ± the sample standard deviation (Liu et al. 2022). Nomenclatural details were submitted to MycoBank. Color terms followed the designations provided by the website ColorHexa (https://www.colorhexa.com).

### ﻿DNA extraction, PCR amplification, and sequencing

DNA extraction was carried out using the M5 Plant Genomic DNA Kit (Mei5 Biotechnology Co., Ltd., China). The extracted DNA was dissolved in 1× TE buffer and stored at –20 °C for later use. PCRs were performed using a Bio-Rad S1000 thermal cycler (Bio-Rad Laboratories, Inc., USA). The primer set ITS5/ITS4 (Vilgalys and Hester 1990; White et al. 1990) was used to amplify the nrITS region, and LR0R/LR5 (Vilgalys and Hester 1990) was used for the nrLSU region. PCRs were conducted in a 25 μL reaction volume containing 2 μL of DNA template, 1 μL of each primer (10 μM), 12.5 μL of 2× Master Mix (Mei5 Biotechnology Co., Ltd., China), and 8 μL of ddH_2_O. PCR amplification conditions for nrITS followed Wannathes et al. (2018), Liu et al. (2022), and Gao et al. (2024), and the conditions for nrLSU followed Yan et al. (2020) and Sui et al. (2023). All DNA sequences were generated by Sangon Biotech (Shanghai) Co., Ltd.

### ﻿Molecular phylogenetic analyses

The newly obtained sequences were submitted to NCBI (https://www.ncbi.nlm.nih.gov). The nrITS and nrLSU sequences were aligned with selected sequences from GenBank and previously published literature (Deng et al. 2024). All sequences used are listed in Table 1. The raw reads of the DNA sequences were processed to obtain consensus sequences using SeqMan 7.1.0 in the DNASTAR Lasergene Core Suite software (DNASTAR Inc., Madison, WI, USA). Sequence alignment was performed using MAFFT 6 (Katoh and Toh 2010), and manual trimming was carried out in MEGA 6 (Tamura et al. 2013). For phylogenetic analyses, newly obtained sequences and additional reference sequences of *Phaeoclavulina* and *Ramaria* species were included in the nrITS and nrLSU dataset (Table 1), with *Gomphusclavatus* (Pers.) Gray designated as the outgroup following Wannathes et al. (2018).

**Table 1.** Specimens used in phylogenetic analysis and their GenBank accession numbers. Newly generated sequences are shown in bold. Voucher numbers marked with “T” indicate holotypes.

Taxonomy	Voucher/strain	Location	References	GenBank Number	
				nrLSU	nrITS
*Gomphusclavatus*	LL 115	China	Kang et al. (2016)	-	KX008988
*G.clavatus*	G 071	USA	Unpublished	AY647207	-
*G.clavatus*	EL 64/03	Sweden	Larsson et al. (2007)	EU118628	EU118628
*Phaeoclavulinaabietina*	U 066	USA	Unpublished	-	KY510818
*P.abietina*	OSC 134649	USA	Unpublished	JX287478	JX310378
*P.abietina*	OSC 140661	USA	Unpublished	-	JX310379
*P.abietina*	OSC 112178	USA	Unpublished	KY354707	-
*P.aeruginea*	MHHNU6887	China	Liu et al. (2022)	ON262781	ON262784
*P.aeruginea*	MHHNU8909 (T)	China	Liu et al. (2022)	ON262782	ON262785
*P.africana*	TENN 39621	USA	Giachini et al. (2010)	AY574653	-
*P.alboapiculata*	AMB 18590 (T)	Italy	Franchi et al. (2022)	MT053248	NR_176722
*P.alboapiculata*	AMB 18585	Italy	Franchi et al. (2022)	MT053242	MT055964
*P.angustata*	BPI2	-	Unpublished	AY577847	-
*P.apiahyna*	LPS 13259	USA	Unpublished	AY577840	-
*P.arcosuensis*	AMB 18532	Italy	Franchi et al. (2022)	MT053207	MT055916
*P.argentea*	AGK 036	USA	Unpublished	-	JQ408231
*P.argentea*	AGK 042	USA	Unpublished	-	JQ408234
***P.aurea***	**BJM 344955 (T)**	**China**	**Present study**	**PQ287860**	**PQ287856**
***P.aurea***	**BJTC L007**	**China**	**Present study**	**PQ287858**	**PQ287853**
*P.bicolor*	MHHNU10702 (T)	China	Deng et al. (2024)	PP800475	PP809798
*P.bicolor*	MHHNU10703	China	Deng et al. (2024)	PP800476	PP809799
*P.campoi*	LPS39622	USA	Unpublished	AY577842	-
*P.capucina*	GH 288	USA	Unpublished	AY577845	-
*P.carovinacea*	AMB 18533 (T)	Italy	Franchi et al. (2022)	-	NR_176719
*P.carovinacea*	AMB 18534	Italy	Franchi et al. (2022)	-	MT055918
*P.caroviridula*	AMB 18535 (T)	Italy	Franchi et al. (2022)	MT053208	NR_177141
*P.caroviridula*	AMB 18536	Italy	Franchi et al. (2022)	MT053209	MT055920
*P.cinnamomea*	MHHNU10376 (T)	China	Liu et al. (2022)	ON262783	ON262786
*P.clavarioides*	ERD-9641	Portugal	Unpublished	-	OQ703628
*P.clavarioides*	PRM:945440	Czech Republic	Kříž et al. (2019)	LR723645	LR723646
*P.cokeri*	TENN 36030	USA	Giachini et al. (2010)	AY574701	-
*P.cokeri*	MA:Fungi:79893	Spain	Martin et al. (2020)	-	MH322666
*P.coniferarum*	AMB 18531 (T)	Italy	Franchi et al. (2022)	NG_088119	NR_176718
*P.corrugata*	SJ99002	-	Larsson et al. (2004)	AY586707	-
*P.curta*	MA-Fungi 48081	Spain	Unpublished	-	AJ408359
*P.curta*	UBC F32034	Canada	Unpublished	-	KX236126
*P.curta*	OSC 8711	USA	Giachini et al. (2010)	AY574713	-
*P.cyanocephala*	TH 9064	Guyana	Unpublished	KT339290	KT339249
*P.cyanocephala*	TENN 37827	USA	Giachini et al. (2010)	AY574710	-
*P.decolor*	FH 1	USA	Unpublished	AY577843	-
*P.decurrens*	257517179	Netherlands	Unpublished	-	OQ586352
*P.echinoflava*	HKAS 45984 (T)	China	Deng et al. (2024)	PP800478	PP809801
*P.echinoflava*	HKAS 45992	China	Deng et al. (2024)	PP800477	PP809800
*P.eumorpha*	GLM:GLM-F116666	Germany	Unpublished	-	OM152300
*P.flaccida*	48020	Spain	Unpublished	-	AJ408390
*P.flaccida*	48076	Spain	Unpublished	-	AJ408371
*P.flaccida*	AMB 18544	Italy	Franchi et al. (2022)	MT053213	MT055926
*P.flaccida*	AMB 18645	Italy	Franchi et al. (2022)	MW092705	MW115424
*P.flaccida*	AMB18643	Italy	Franchi et al. (2022)	MW092707	MW115426
***P.fulva***	**BJTC C274 (T)**	**China**	**Present study**	**PQ287857**	**PQ287852**
***P.fulva***	**BJTC ZH0015**	**China**	**Present study**	-	**PQ287854**
***P.fulva***	**BJTC ZH1138**	**China**	**Present study**	**PQ287859**	**PQ287855**
*P.gigantea*	FH 109	USA	Giachini et al. (2010)	AY574703	-
*P.grandis*	BR079158-06	USA	Giachini et al. (2010)	AY574678	-
*P.guyanensis*	FH84	USA	Giachini et al. (2010)	AY574706	-
*P.insignis*	FH104	USA	Giachini et al. (2010)	AY574704	-
*P.jilinensis*	MHHNU9149	China	Deng et al. (2024)	PP800479	PP809802
*P.jilinensis*	MHHNU9164	China	Deng et al. (2024)	PP800480	PP809803
*P.jilinensis*	MHHNU10504 (T)	China	Deng et al. (2024)	PP800481	PP809804
*P.liliputiana*	3281	Mexico	González et al. (2020)	MT214488	-
*P.liliputiana*	3533	Mexico	González et al. (2020)	MT214489	-
*P.liliputiana*	3266 (T)	Mexico	González et al. (2020)	MT214490	-
*P.liliputiana*	3563	Mexico	González et al. (2020)	MT214491	-
*P.longicaulis*	TENN 33826	USA	Giachini et al. (2010)	AY574700	-
*P.macrospora*	AMB 18614	Italy	Franchi et al. (2022)	-	MT452510
*P.minutispora*	GT21030	Belgium	Unpublished	OQ746395	OQ749903
*P.minutispora*	LD5028	Belgium	Unpublished	OQ746341	OQ729761
*P.murrillii*	AH:48382	Spain	Martín et al. (2020)	-	MH322683
*P.mutabilis*	TENN 39893	USA	Unpublished	AY577838	-
*P.myceliosa*	ANT057-QFB28646	Canada	Unpublished	-	MN992499
*P.nigricans*	AMB 18589	Italy	Franchi et al. (2022)	MT053247	MT055970
*P.ochracea*	S 1	USA	Unpublished	AY577850	-
*P.ochracea*	AMB 18542	Italy	Franchi et al. (2022)	MT053211	MT055924
*P.ochraceovirens*	OSC 23475	USA	Giachini et al. (2010)	AY574714	-
*P.pancaribbea*	TENN31836	USA	Giachini et al. (2010)	AY574707	-
*P.pseudozippelii*	BBH 43576	Thailand	Wannathes et al. (2018)	MG214662	MG214660
*P.pseudozippelii*	BBH 43575 (T)	Thailand	Wannathes et al. (2018)	MG214663	MG214661
*P.quercusilicis*	MA-Fungi 47984	Spain	Unpublished	-	AJ408382
*P.roellinii*	PRM:945446	Czech Republic	Kříž et al. (2019)	-	LR723648
*P.subclaviformis*	BR079159-07	-	Giachini et al. (2010)	AY574679	-
*P.subdecurrens*	AMB 18548	Italy	Franchi et al. (2022)	MT053217	MT055930
*P.tropicalis*	NY551	USA	Unpublished	AY577841	-
*P.yunnanensis*	HKAS 127150	China	Zheng et al. (2024)	PQ376602	OQ755411
*P.yunnanensis*	HKAS 128154	China	Zheng et al. (2024)	PQ376603	OQ755412
*P.zealandica*	PDD43383	-	Unpublished	AY577849	-
*P.zippelii*	FH2	USA	Unpublished	AY577844	-
*Ramariaadmiratia*	TENN 69114	USA	Petersen et al. (2014)	KJ416134	-
*R.admiratia*	TFB 14450 (T)	USA	Petersen et al. (2014)	-	KJ416133
*R.aurantiisiccescens*	OSC 65703	USA	Unpublished	EU669298	EU669247
*R.aurantiisiccescens*	OSC 130871	USA	Unpublished	JX287480	JX310388
*R.botrytis*	AMB 18201 (T)	Italy	Franchi et al. (2022)	NG_241889	NR_189799
*R.botrytis*	GM19124	Argentina	González et al. (2022)	OP177872	OP177708
*R.calvodistalis*	TENN 69095	USA	Petersen et al. (2014)	KJ416135	-
*R.calvodistalis*	TENN 69095 (T)	USA	Petersen et al. (2014)	-	KJ416132
R.cf.celerivirescens	AGK 072	USA	Unpublished	-	JQ408243
*R.foetida*	AGK 058	USA	Unpublished	JQ408239	-
*R.gracilis*	OSC 134659	USA	Unpublished	JX287487	JX310399
*R.gracilis*	OSC 112168	USA	Unpublished	KY354718	KY354745
*R.largentii*	OSC 67012	USA	Unpublished	KP637058	KP658130
*R.largentii*	OSC 140726	USA	Unpublished	JX287489	-
*R.luteovernalis*	MCVE 28662	Italy	Franchi et al. (2015)	-	KT357476
*R.luteovernalis*	MCVE 28637 (T)	Italy	Franchi et al. (2015)	-	KT357471

To estimate maximum likelihood (ML) gene trees, RAxML 7.4.2 Black Box software was used (Stamatakis 2006; Stamatakis et al. 2008; Zhou and Hou 2019; Zhou et al. 2021), employing a GTRGAMMAI site substitution model (Guindon et al. 2010). Branch support was assessed with 1000 bootstrap (BS) replicates (Hillis and Bull 1993). Bayesian Inference (BI) analysis was conducted using MrBayes 3.1.2 (Ronquist and Huelsenbeck 2003), with a Markov chain Monte Carlo (MCMC) algorithm (Rannala and Yang 1996). The best-fit substitution model was determined using MrModeltest 2.3 (Zhou and Hou 2019; Zhou et al. 2021, 2022). The selected models were SYM + G for nrITS and SYM + I + G for nrLSU. Two MCMC chains were run for 10,000,000 generations, terminating when the average standard deviation of split frequencies fell below 0.01. Trees were sampled every 1000 generations, with the first 25% discarded as burn-in. Significant Bayesian posterior probabilities (pp) were calculated for branches in the resulting majority-rule consensus trees. The analyses yielded relatively stable topologies, and clades with high pp values reflected the phylogenetic relationships among species (Posada and Crandall 1998).

## ﻿Results

### ﻿Molecular phylogeny

**Figure 1.**
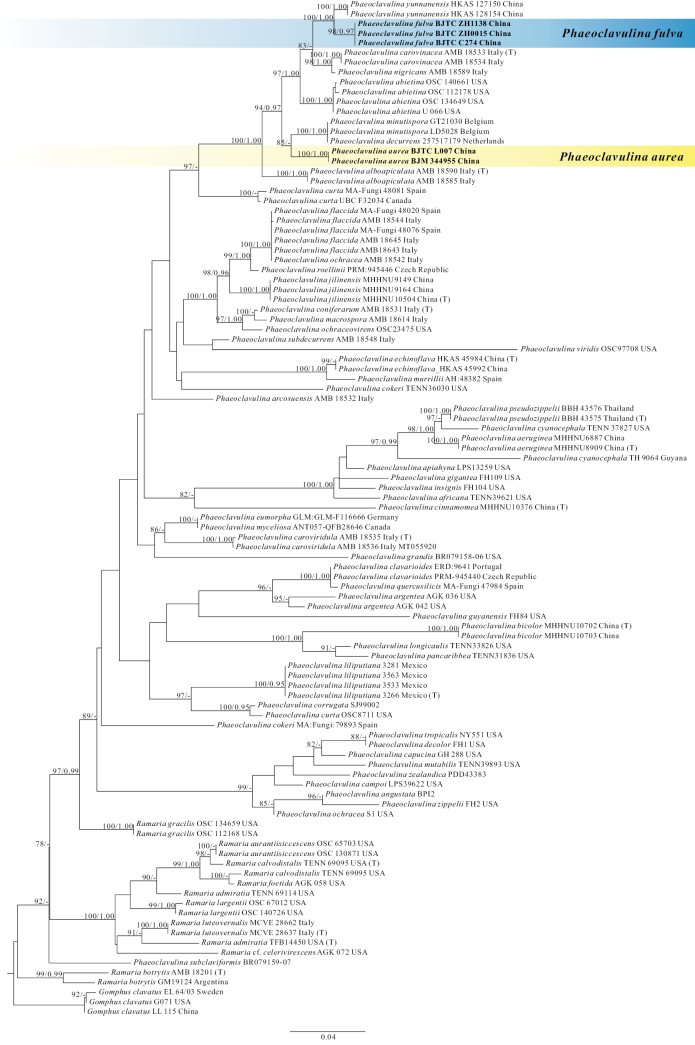
Phylogenetic tree generated from an ML analysis based on nrITS-nrLSU sequences. Numbers representing maximum likelihood bootstrap support (MLBS ≥ 75%, left) and significant Bayesian posterior probability (BPP ≥ 0.95, right) are indicated above the nodes. Novel sequences are printed in bold. Voucher specimens and localities where the specimens were collected are provided behind the species names.

A total of nine sequences, including five for nrITS and four for nrLSU, were newly generated in this study. The nrITS and nrLSU datasets were compiled to investigate the phylogenetic position of the new species in *Phaeoclavulina*. The combined nrITS–nrLSU dataset included 146 sequences (69 for nrITS and 77 for nrLSU), representing 102 samples. The concatenated alignment contained 840 characters, including gaps. Maximum Likelihood (ML) and Bayesian Inference (BI) analyses yielded highly similar tree topologies; therefore, only the tree inferred from the ML analysis is shown (Fig. 1).

The tree topology is consistent with that of Zheng et al. (2024). In the phylogenetic reconstruction, each of the two new species, *P.aurea* and *P.fulva*, formed a distinct monophyletic lineage within the *Phaeoclavulina* clade (Fig. 1). The specimens of *P.aurea* (BJM 344955 and BJTC L007) and *P.fulva* (BJTC C274 and BJTC ZH1138) were identified as the sister species of *P.yunnanensis*, with strong statistical support (MLB = 100%, BPP = 1.00). *Phaeoclavulinaaurea* (BJM 344955 and BJTC L007) formed a clade with *P.minutispora* and *P.decurrens*, although this relationship was supported by a relatively lower value (MLB = 85%, BPP = 0.83). In conclusion, the ML and BI analyses of the nrITS–nrLSU dataset support the recognition of two new species of *Phaeoclavulina* from China, viz. *P.aurea* and *P.fulva*.

### ﻿Taxonomy

#### ﻿ Phaeoclavulina aurea

Taxon classificationFungiGomphalesGomphaceae

Y. Gao, X. Tong, & C.L. Hou sp. nov.

E6FF7311-A747-5C13-B2A0-355B5531A117

855628

Figs 2A–C , 3

##### Diagnosis.

*Phaeoclavulinaaurea* differs from the known species of *Phaeoclavulina* in its pale yellow to golden yellow basidiomata, tips brownish black when age, truncate spines basidiospores 4.7–6.3 × 3.0–3.7 μm, basidia 25–50 × 4.6–7.4 μm, clamp connections present.

**Figure 2.**
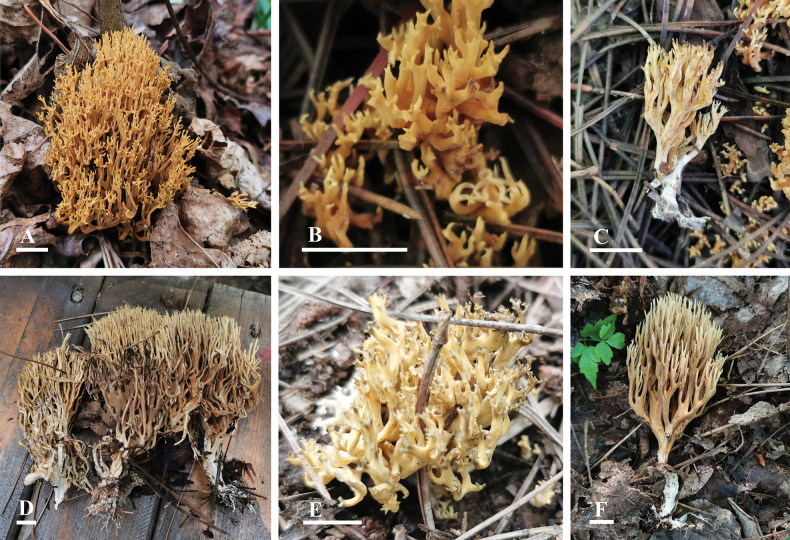
Morphology of Basidiomata. A–C. *Phaeoclavulinaaurea* (A, B. BJM 344955 holotype; C. BJTC L007); D–F. *Phaeoclavulinafulva* (D. BJTC C274 holotype; E. BJTC ZH0015; F. BJTC ZH1138). Scale bars: 1 cm.

##### Etymology.

The epithet “*aurea*” refers to the yellow to golden yellow basidiomata.

**Figure 3.**
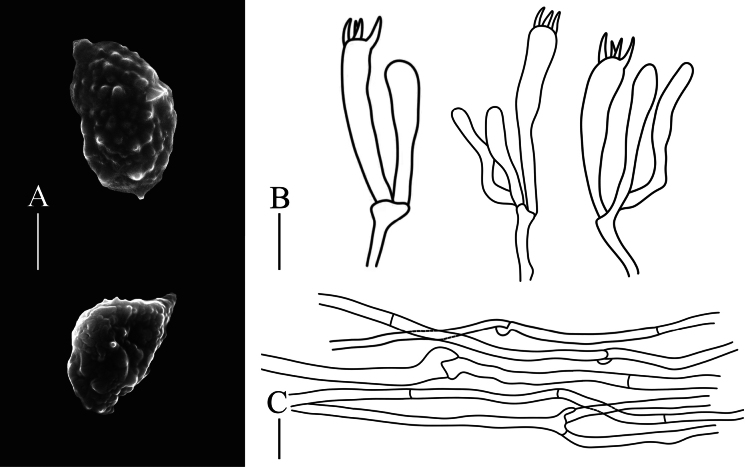
Microscopic characteristics of *Phaeoclavulinaaurea*: A. Basidiospores; B. Basidia; C. Tramal hyphae. Scale bars: 20 µm (A); 10 µm (B, C).

##### Type.

China • Beijing, Yanqing District, Songshan, 40°30'28"N, 115°54'E, elev. 869 m, 16 Aug. 2023, H. Zhou, Y. Gao & X. Tong (BJM 344955). GenBank nrITS: PQ287856, nrLSU: PQ287860.

##### Description.

Basidiomata coralloid, solitary or scattered; individual basidiomata 3–8 cm tall, 2–5.5 cm wide across branches. Stipe 0.4–0.9 cm tall, 0.3–0.5 cm wide, subclavate to flattened, snow-white rhizomorphic strands near the ground, branches di- or tri-dichotomous, generally 3–4 times, cylindrical, pale yellow (#ffdd9a) to golden yellow (#ffc44d); as maturity deepens, tips blunt and short with brownish black (#473826), stipe surface white (#ffffff) to pale yellow (#ffdd9a). No color change when bruised. Odor and taste not recorded.

Basidiospores [70/2/2] (4.4–)4.7–6.3(− 6.7) ×3.0–3.7(− 4.2) μm, *Q* = 1.4–1.9(− 2.1), *Q_m_* = 1.66 ± 0.16, ellipsoid, pale yellow to golden yellow in KOH, with thick wall and cyanophilic ornamentation in cotton blue; when seen with SEM, conical, truncate spines up to 0.6 µm high; with oleiferous guttule contents. Hilar appendixes acuminate (up to 1.1 µm in length). Basidia 25–50 × 4.6–7.4 µm, clavate, 3–4 sterigmata occur per basidium, 3.6–6.0 μm long, and cornute, clamped. Basidioles abundant, subclavate to subcylindrical. Tramal hyphae in the stipe smooth, thin-walled, hyaline in KOH, 2.3–5.8 μm wide; tramal hyphae in branch with hyaline and thin-walled, 1.8–4.0 μm wide; clamp connections abundant, H-connections present. Hyphal system monomitic. Cystidia absent.

##### Habit, habitat, and distribution.

Solitary or gregarious caespitose in humus layers on soils in mixed coniferous and broad-leaved forests, associated with *Pinus* L. Basidiomata generally occur in August; currently known only from Beijing, China.

##### Additional specimens examined.

China • Beijing, Huairou District, Hongluo Temple, 40°22'26"N, 116°37'26"E, elev. 153 m, 13 Aug. 2019, J.Q. Li, X.Y. Shen & R.T. Zhang (BJTC L007).

##### Notes.

*Phaeoclavulinaaurea* is morphologically similar to *P.cinnamomea*, which was originally described from China. However, *P.cinnamomea* produced cinnamon-to-salmon-orange basidiomata and relatively large basidiospores (12–15 × 5–7 µm) and basidia (40–60 × 8–12 µm) compared *P.aurea* (Liu et al. 2022). *Phaeoclavulinaangustata* (Lév.) Giachini (2011: 189) also has crowded branches, but the color of that is pale pink, and all parts soon turned sordid vinaceous on bruising, and the cyanophilic spines of basidiospores 1–3.5 µm tall (Giachini 2004). *Phaeoclavulinaglaucoaromatica* (R.H. Petersen) Giachini (2011: 193) has similar basidiomata with *P.aurea*, which doesn’t have molecular data. But *P.glaucoaromatica* differs from *P.aurea* by its branches becoming watery where handled and then changing color to olive-green; basidiospores are larger (8.2–11.1 × 4.4–5.5 µm) (Petersen 1981). *Phaeoclavulinavinaceipes* (Schild) Giachini (2011: 195) has the same branch color as *P.aurea*, which also doesn’t have molecular data. But *P.vinaceipes*’s stipe context bruising violet when exposed or handled, per basidium occur 4 sterigmata, and basidiospores are wider (4.8–8 × 2.7–3.8 µm) (Schild 1990). *Phaeoclavulinaaurea* is phylogenetically close to *P.abietina*, *P.alboapiculata* Franchi & M. Marchetti (2020: 1), and *P.minutispora* Franchi & M. Marchetti (2020: 3) (Fig. 1), but the stipes of *P.abietina* quickly turned deep blue-green when handled or confined (González-Ávila et al. 2020); the branches of *P.alboapiculata* are with white tips when young, and the white context gradually turns to wine when exposed to the air, and it has smaller basidiospores (4.1–5.5 × 2.5–3.4 µm) and basidia (22–30 × 5.0–6.5 µm) than *P.aurea* (Index Fungorum 2024); *P.minutispora* can be distinguished by its basidiomata surface, which gradually shows dark brown pigmentation when touched, white flesh turns maroon rapidly when exposed to air, and producing relatively small basidiospores (3.7–5.0 × 2.5–3.6 µm) and basidia (22–30 × 5–6.5 µm) (Index Fungorum 2024).

#### ﻿ Phaeoclavulina fulva

Taxon classificationFungiGomphalesGomphaceae

Y. Gao, X. Tong, & C.L. Hou sp. nov.

CEA9616D-989F-5274-ACBE-217BC04E3B65

855499

Figs 2D–F , 4

##### Diagnosis.

*Phaeoclavulinafulva* differs from the known species of *Phaeoclavulina* in its dirty orange basidiomata, stipe surface pale yellow, color lightens towards tips, rounded warts basidiospores 4.8–7.8 ×2.8–3.8 μm, basidia 22–43 × 4.3–7.3 μm.

**Figure 4.**
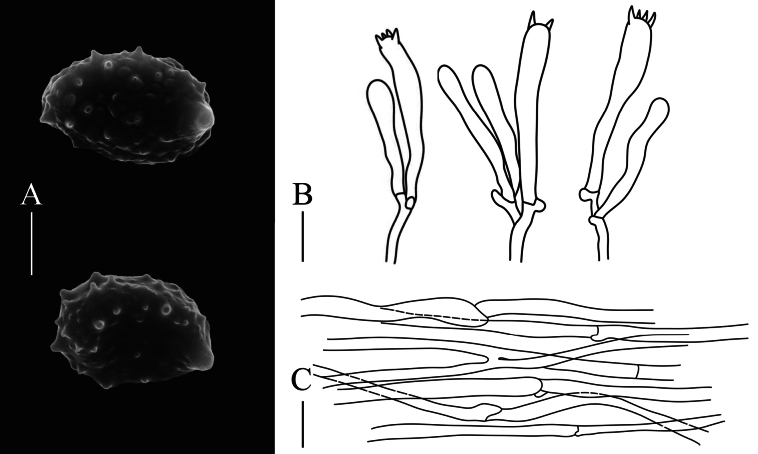
Microscopic characteristics of *Phaeoclavulinafulva*: A. Basidiospores; B. Basidia; C. Tramal hyphae. Scale bars: 20 µm (A); 10 µm (B, C).

##### Etymology.

The epithet “*fulvus*” refers to the dirty orange to yellowish-brown basidiomata.

##### Type.

China • Tianjin, Jizhou District, Limutai Scenic Spot, 40°12'47"N, 117°2'7"E, elev. 658 m, 21 Aug. 2020, G.Q. Cheng, R.T. Zhang & C.L. Hou (BJTC C274). GenBank nrITS: PQ287852, nrLSU: PQ287857.

##### Description.

Basidiomata coralloid, solitary or scattered; individual basidiomata 4.2–12.5 cm tall, 4.5–10 cm wide across branches. Stipe 1.2–3.8 cm tall, 0.3–0.5 cm wide, subclavate to flattened, snow-white rhizomorphic strands near the ground, branches dichotomous, generally 3–4 times, subcylindrical, dirty orange (#d5995b) to yellowish-brown (#a27f16), tips slightly acute, stipe surface pale yellow (#fff7dc), color lightens towards tips. No color change when bruised. Odor and taste not recorded.

Basidiospores [90/4/3] (4.3–)4.8–7.8 × (2.5–)2.8–3.8(− 4.2) μm, *Q* = 1.5–2.2(− 2.37), *Q_m_* = 1.89 ± 0.19, elongate-ellipsoid, pale yellow to golden yellow in KOH, with thick wall and cyanophilic ornamentation in cotton blue; when seen with SEM, conical, truncate warts up to 0.6 µm high; with oleiferous guttule contents. Hilar appendixes acuminate (up to 1.0 µm in length). Basidia 22–43 × 4.3–7.3 µm, clavate, 2–4 sterigmata occur per basidium, 1.8–7.0 μm long, and cornute, clamped. Basidioles abundant, subclavate to subcylindrical. Tramal hyphae in the stipe smooth, thin-walled, hyaline in KOH, 2.2–7.5 μm wide; tramal hyphae in branch with hyaline and thin-walled, 2.5–6.5 μm wide; clamp connections abundant, H-connections present. Hyphal system monomitic. Cystidia absent.

##### Habit, habitat, and distribution.

Solitary or gregarious caespitose in humus layers on soils in evergreen coniferous forests and deciduous broad-leaved forests, associated with *Pinus* L. and *Populus* L. Basidiomata generally occur from August; currently known only from Tianjin and Beijing, China.

##### Additional specimens examined.

China • Beijing, Huairou District, Hongluo Temple, 40°22'34"N, 116°37'28"E, elev. 109 m, 13 Aug. 2019, H. Zhou, G.Q. Cheng & C.L. Hou (BJTC ZH0015); China • Beijing, Huairou District, Erdaogou, 40°52'36"N, 116°31'23"E, elev. 780 m, 24 Aug. 2020, H. Zhou, X.Y. Shen & X.B. Huang (BJTC ZH1138).

##### Notes.

*Phaeoclavulinafulva* generally shares similar branching with *P.sikkimia*, *P.cokeri*, and *P.articulotela* (R.H. Petersen) Giachini (2011: 190). *Phaeoclavulinasikkimia* and *P.articulotela* lack molecular data (Petersen 1981). However, *P.sikkimia* differs from *P.fulva* in its relatively large basidiospores (6.5–9 × 4.5–6 µm) and basidia (60–70 × 7.5–9 µm) and in the presence of cyanophilic warts up to 2 µm tall (Giachini 2004). *P.cokeri* has cinnamon- to pink-yellow branches that become orange or dark red-brown, with pale orange tips; it also features strongly cyanophilic basidiospore spines 1–2 µm tall and relatively large basidiospores (9–16 × 4–7.5 µm) and basidia (45–80 × 7.5–12 µm) (Giachini 2004). *P.articulotela* has orange to dark orange basidiomata and relatively large basidiospores (6–9.5 × 3–5 µm) (Giachini 2004). Phylogenetically, *P.fulva* is related to *P.abietina*, *P.carovinacea* Franchi & M. Marchetti (2020: 2), *P.nigricans* E. Campo, Franchi & M. Marchetti (2020: 4), and *P.yunnanensis* in the analyses of the multilocus datasets, respectively (Fig. 1). However, *P.abietina* has a stipe that is olive-ochraceous to dull ocher upward, quickly turning deep blue-green when handled or confined; its branches are yellow-ocher to dull ocher when fresh or somewhat greenish-ocher (González-Ávila et al. 2020). *Phaeoclavulinacarovinacea* differs from *P.fulva* in its white flesh, which turns reddish brown when exposed to air (Index Fungorum 2024). *Phaeoclavulinanigricans* has basidiomata whose surface gradually develops a burgundy coloration when touched, and its light cream flesh turns black rapidly upon exposure to air (Index Fungorum 2024). *Phaeoclavulinayunnanensis* is characterized by its yellow basidiomata, with the tips showing a slight greenish-grey tint, and relatively small basidia (20–40 × 3–5 µm) (Zheng et al. 2024). The species also differs from the other new species described in this study, *Phaeoclavulinaaurea*, in both basidiomata color and basidiospore morphology. *Phaeoclavulinaaurea* has pale yellow to golden yellow basidiomata and basidiospores with truncate spines.

## ﻿Discussion

The ornamentation of spores holds paramount significance in differentiating species within *Phaeoclavulina*. Previous studies have classified spore ornamentation in R.subg.Echinoramaria into five distinct types (Petersen 1981; Villegas et al. 2005). Volcanic spines, a distinctive form of spore ornamentation, are also evident in distinguishing *Phaeoclavulina* species, such as *P.pancaribbea* (R.H. Petersen) Giachini (2011: 194) (Petersen 1981; Giachini and Castellano 2011) and *P.zealandica* (R.H. Petersen) Giachini (2011: 195) (Petersen 1988; Giachini and Castellano 2011). The new species *P.aurea* and *P.fulva* described in this study also possess truncate warts or spines. However, it is noteworthy that both *P.pancaribbea* and *P.zealandica* have larger spores than the newly discovered *P.aurea* and *P.fulva*.

Some species within *Phaeoclavulina* exhibit host specificity. For example, *P.yunnanensis* has only been found under *Quercus* sp. (Zheng et al. 2024), *P.cyanocephala* is exclusively associated with *Abies* sp. (Estrada-Torres 2007), and the new species *P.aurea* discovered in this study has only been found under *Pinus* sp. This host specificity may serve as one of the diagnostic characteristics for species identification within *Phaeoclavulina*. Additionally, numerous species remain undiscovered globally, particularly in understudied forest ecosystems with favorable environmental conditions (Exeter et al. 2006), and many endemic taxa are still awaiting documentation. Therefore, further exploration of these habitats, combined with molecular and ecological studies, is essential to uncover the full diversity and host associations of *Phaeoclavulina* species.

Previous studies have indicated that clavarioid fungi, including *Phaeoclavulina*, are highly diverse and widely distributed across the globe (Corner 1950). However, to date, only 22 species of *Phaeoclavulina* have been described from China, despite the country’s vast territory, complex climate, diverse habitats, and rich species resources, all of which contribute to its exceptionally high fungal diversity (Miller et al. 1991). This gap may be attributed to the limited number of collections. In this study, two new species of *Phaeoclavulina* from North China were described using nrITS–nrLSU phylogenetic analyses (Fig. 1) and macrofungal morphological examination, thereby contributing to the current understanding of the species diversity within this genus. Moreover, specimen collection and field investigation were conducted only in August, suggesting that additional *Phaeoclavulina* species may yet be discovered in this study area.

## Supplementary Material

XML Treatment for Phaeoclavulina aurea

XML Treatment for Phaeoclavulina fulva

## ﻿Additional information

### Use of AI

No use of AI was reported.

### Author ORCIDs

Xin Tong https://orcid.org/0000-0002-2091-6636

Yue Gao https://orcid.org/0009-0001-0831-3656

Hai-Qi Wang https://orcid.org/0009-0004-3388-6263

Hao Zhou https://orcid.org/0000-0002-4869-2187

Cheng-Lin Hou https://orcid.org/0000-0001-8162-5560

### Data availability

All of the data that support the findings of this study are available in the main text.
